# Carotid Doppler findings among patients admitted with stroke in two tertiary care facilities in Uganda: A Hospital-based Cross-sectional Study

**DOI:** 10.21203/rs.3.rs-2800534/v1

**Published:** 2023-04-28

**Authors:** Jonathan Walubembe, Isaac Ssinabulya, Aloysius G. Mubuuke, Mark Mohan Kagwa, Deborah Babirye, Jerom Okot, Felix Bongomin, Miriam Nakku, Diana Okello Ongom, Faith Ameda

**Affiliations:** Makerere University; Makerere University; Makerere University; McMaster University Hamilton; Makerere University; Gulu university; Gulu university; Makerere University; Makerere University; Makerere University

**Keywords:** Carotid Doppler, stroke, atherosclerosis, lipoprotein cholesterol, carotid intima media thickness, Uganda

## Abstract

**Background:**

Carotid Atherosclerotic Disease (CAD) Doppler findings including carotid artery stenosis, thickened intima media thickness, and high-risk atherosclerotic plaques are associated with increased risk of stroke and symptomatic cerebrovascular disease. However, few studies have explored these Doppler characteristics among stroke patients in Africa. This study, therefore, investigates these carotid artery Doppler characteristics among stroke patients in Uganda.

**Methods:**

A hospital-based cross-sectional study of 95 stroke patients attending two national referral hospitals in Uganda between March and July 2022. Following the caption of their sociodemographic and clinical characteristics, they underwent Doppler sonography of the extracranial carotid arteries using a standard carotid Doppler protocol. Multivariate logistic regression was used to determine factors associated with abnormal carotid Doppler parameters (i.e., carotid intima-media thickness, carotid stenosis).

**Results:**

The mean age of the study participants was 61 ± 13 years with 60% (57/95) of the participants being male. Most participants had an ischemic stroke (67%), hypertension (76.4%), and used alcohol (58.9%). The prevalence of significant carotid stenosis in participants with ischemic stroke was 12.5% (8/64) (i.e., 7.8% had severe carotid stenosis and 4.7% had moderate stenosis). The prevalence of high carotid intima media thickness (CIMT) and atherosclerotic plaques were 31.6% (30/95) and 26.3% (25/95), respectively. Most atherosclerotic plaques were echogenic. Age above 60 years (adjusted odds ratio [aOR] = 5.2, 95% Confidence Interval [CI]:1.97–14; *p* < 0.010), high low-density lipoprotein cholesterol (aOR = 4.2, 95% CI: 1.29–8.79; *p* = 0.013) increased the likelihood of having abnormal CIMT.

**Conclusion:**

The burden of carotid atherosclerotic disease is increasing among stroke patients in Uganda. Large-scale epidemiological studies are needed to further profile the disease in high risk populations.

## Introduction

Stroke is defined as rapidly developing clinical signs of focal (or global) disturbance of cerebral function, with symptoms lasting 24 hours or longer or leading to death, with no apparent cause other than of vascular origin [1]. Globally, stroke is the second most common cause of death, contributing to approximately 11.6% % of all deaths, and the third leading cause of disability [2]. In Uganda, stroke is estimated to be one of the top five causes of adult deaths, accounting for approximately 3.7% of all hospital admissions among adults [3] and 27% of all admissions to the neurology ward of Mulago National Referral Hospital (MNRH) [4]. All-stroke mortality from the available Ugandan hospital-based studies is estimated to be between 30 and 40% in one month [5, 6].

Large vessel atherosclerotic cerebrovascular disease is an important cause of stroke and produces a higher risk of early recurrent ischemia than any other stroke subtype [7]. Patients with carotid stenosis (< 75%), the annual incidence of stroke is 1.3% [8, 9] while patients with more than 70% carotid artery stenosis have a stroke rate of 28% at 18 months [9]. Carotid artery stenosis can lead to stroke by multiple mechanisms including: embolism, thrombotic occlusion, dissection, or hypoperfusion [10]. On the other hand, carotid stenosis is considered a marker of systemic atherosclerosis and has a significant association with myocardial infarction [11].

Carotid Intima-Media Thickness (CIMT) is an established non-invasive predictor of the incidence of future ischemic stroke, as it accurately reflects the early stages of atherosclerosis and cardiovascular risk [12]. A thickening of the CIMT complex not only reflects local alterations in the Common Carotid Artery (CCA), but also corresponds to generalized atherosclerosis [13, 14]. A positive association between CIMT and incident stroke of all types has been reported in epidemiological studies [15–18]. Several factors have been reported to be associated with an increase in CIMT including chronic use of alcohol, history of smoking, diabetes, hypertension, increase in age, elevated LDL cholesterol, male gender, among others [19–24].

The Doppler evaluation of carotid arteries is a safe and non-invasive examination in the early evaluation of extracranial insufficiency. Carotid atherosclerotic parameters that have been explored include carotid stenosis, CIMT, and atherosclerotic plaques. Besides estimating the degree of stenosis, sonography is able to characterize plaques and identify those with a higher stroke risk [25]. Accurate diagnosis of hemodynamically significant stenosis through assessment of the related gray scales and Doppler parameters is critical to identify patients who would benefit from surgical or medical intervention. The North American Symptomatic Carotid Endarterectomy Trial (NASCET) and European Carotid Surgery Trialists (ECST) collaborative group showed a benefit of carotid endarterectomy for recently symptomatic patients with internal carotid lumen diameter narrowing of 70% or more [9].

Studies published a decade ago showed prevalence of carotid stenosis to be uncommon in stroke patients in sub-Saharan Africa (SSA) [26–28]. This is likely to change as the population advances in age through the epidemiologic and demographic transition, increasing prevalence of hypertension [29] and without adequate risk factor prevention. The few recent studies in SSA Africa document an increasing prevalence of carotid atherosclerotic disease [20, 30]. In many low-income settings, there is limited current baseline evidence of carotid atherosclerotic disease in patients admitted with stroke. The purpose of the present study, therefore, was to establish baseline evidence about the current prevalence and associated factors of the carotid atherosclerotic disease in Uganda – a low-income country in SSA.

## Methods and materials

It was a hospital-based cross-sectional study involving 95 consenting adults (> 18 years) admitted and diagnosed with a stroke in the vascular territory of internal carotid artery. The study took place at two public tertiary hospitals in Uganda (Kiruddu National Referral Hospital [KNRH] and Mulago National Referral Hospitals [MNRH]). Patients with trauma or brain neoplasms were excluded from the study. The general standard of care for patients suspected of having a stroke in the two hospitals includes clinical assessment, laboratory workup, and CT scan to inform clinical management. Neurologists review the patients and adjust treatment accordingly. Patients who improve are discharged with routine reviews in the neurology clinics of MNRH and KNRH.

### Study procedure

Study participants admitted to the neurology wards of the two hospitals were identified by the research assistant with the help of the attending. Consecutive sampling was used after file reviews, with study participants meeting the inclusion criteria being recruited into the study. Informed consent was obtained from the patient or his guardian. Following the consent, a questionnaire was filled from the patient’s medical file/records; whose contents included: (i) sociodemographic characters (age in years, gender), (ii) atherosclerotic risk factors documented in patients’ hospital records (history of; hypertension and/or treatment for hypertension, diabetes mellitus, and smoking), and others (serum lipid profile, fasting blood sugar level, and blood pressure - baseline laboratory tests done on patients’ admission). Lastly, a Doppler sonography of the carotid arteries was performed using a portable ultrasound machine.

### Equipment and sonographic technique

Doppler sonography was performed using a portable ultrasound machine, EDAN U60 series (2020, shanghai Holdings Inc) with a linear array transducer of 5–10MHz. Doppler Sonography of the extracranial carotid arteries was performed on every eligible patient within one week of recruitment into the study. To eliminate interobserver variation, the same radiology resident, with additional training in use of Doppler for the current study, performed all sonographic examinations under the supervision of a senior radiologist with experience in carotid Doppler ultrasound, who routinely reviewed and confirmed the findings.

The carotid arteries on each side of the neck were examined with the patients in the conventional position (patient lying supine, neck slightly extended with the head turned away from the side to be examined) to adequately visualize the vessels. According to published guidelines the carotid arteries were examined in sequence beginning with the common carotid, carotid bifurcation and internal carotid arteries [31, 32].

On gray scale, presence or absence of atheromatous plaque, location of plaque, and plaque characteristics such as echo pattern, calcification, were documented. The identified atheromatous plaques were characterized and documented in five types according to modified Gray-Weale classification as follows: Type 1 - uniformly echolucent, Type 2 - predominantly echolucent, Type 3 - predominantly echogenic, Type 4 - uniformly echogenic, and Type 5 - plaques that could not be classified owing to heavy calcification and acoustic shadows [33]. Type 1 and 2 were then labelled echolucent, and type 3–5 plaques as echogenic atherosclerotic plaques. The height and length of the plaques were also measured. Atheromatous plaques were defined according to the Mannheim consensus definition as a focal structure encroaching into the arterial lumen of at least 0.5 mm or 50% of the surrounding CIMT value, or demonstrates a thickness > 1.5 mm as measured from the media-adventitia interface to the intima-lumen interface [34]. Color Doppler was then used then applied to detect turbulent flow at areas of stenosis.

CIMT measurements (main outcome variable) were made within a region free of plaque with a clearly identified double-line pattern on the far wall of the CCA at least 10 mm below its end. Proximal, mid, and distal measurements were taken, and a mean obtained. The intima-media thickness was defined as the distance between the inner echogenic line representing the intima-blood interface and the outer echogenic line representing the adventitia-media junction. Mannheim Carotid Intima-Media Thickness and Plaque Consensus recommendations [34] were applied to identify patients with abnormal CIMT using the average of values obtained on each CCA. A CIMT thickness of 1mm or more was considered high.

For Doppler spectral analysis, the PSV and EDV were measured in the proximal, mid, and distal portion of the common carotid artery and proximal internal carotid artery beyond the carotid bulb, sequentially on both sides. For velocity recordings, a sample volume of 1.0 mm was used with the cursor in the center of the vessel on a longitudinal image, and a Doppler angle below 60° was used, with the cursor as parallel to the vessel lumen as possible. The degree of stenosis determined at gray-scale and spectral Doppler Ultrasound was stratified into the categories of normal (no stenosis), < 50% stenosis, 50–69% stenosis, ≥ 70% stenosis to near occlusion, and total occlusion as per the consensus criteria of radiologists in Ultrasound guidelines [35].

### Data analysis

Data was analyzed using STATA Version 17.0 in three stages: univariate, bivariate and multivariate analyses. In the univariate analysis, categorical data was summarized using frequencies and percentages; while continuous baseline variables were reported as mean (standard deviation [*SD*]) for normally distributed variables, otherwise median (interquartile range, (IQR) was reported. At bivariate analysis, association between categorical variables and abnormal carotid Doppler parameters (i.e., carotid intima-media thickness [CIMT], carotid stenosis, and atherosclerotic plaques) were assessed using Pearson’s Chi-square or Fisher’s exact tests as appropriate. For numerical variables, independent sample student t-test for parametric variables and Mann-Whitney U tests were used to assess associations for non-parametric variables. Candidate variables with *P* values less than 0.2 in the bivariate analysis were selected for multivariate logistic regression analysis. The backward stepwise elimination was applied to determine the statistical significance of each independent variable. A *p*-value of < 0.05 was considered significant.

## Results

### Clinical and social demographic characteristics

The mean age was 61 (*SD* = 13) years. Majority of the study participants were male (60%). There was a history of diabetes in 19 (20%) study participants. Among the participants 67.4% (64/95) had a diagnosis of ischemic stroke. Among the modifiable cardiovascular risk factors, a history of smoking was documented in 19 (20%) study participants. Hypertension was common among the study participants with 76.4% (n = 73) of the study participants being hypertensive. (Table 1).

### Carotid intima media thickness

The mean carotid intima thickness in the carotid arteries was 0.88(interquartile range, 0.75–1.07). The CIMT in the left internal carotid arteries and right internal carotid artery were 0.87 (0.73–1.09, IQR), and 0.87 (0.72–1.03 IQR) respectively. The prevalence of thickened CIMT in the study participants was 31.6% (30/95). The prevalence of CIMT in the left common carotid (35.8%) was higher than in the right common carotid artery (32.6%) (Table 2).

### Frequency and location of carotid atherosclerotic plaques

Twenty-five participants (25/95) had carotid atherosclerotic plaques, of which the majority were echogenic (60%). Sixty percent (n = 16) of the atherosclerotic plaques were located at the carotid bulb while plaques in the common carotid artery were present in 8% of the participant (Table 3).

### Prevalence of carotid stenosis in ischemic stroke

**Figure 1:**
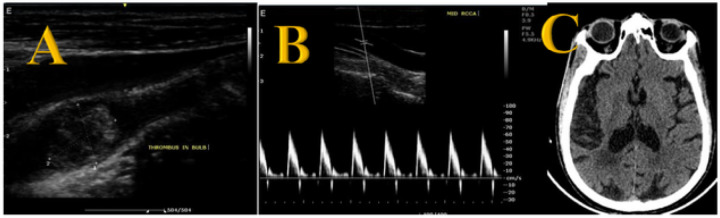
severe carotid artery stenosis Sagittal ultrasound image (Figure IA) of the right common carotid artery in a 67-year-old male with uncontrolled hypertension showing a plaque in the right common artery bulb causing severe carotid stenosis. A spectral doppler proximal to the obstruction (figure 1B) shows a thud waveform. A corresponding right temporal lobe ischemic infarct is seen (Figure IC)

**Figure 2:**
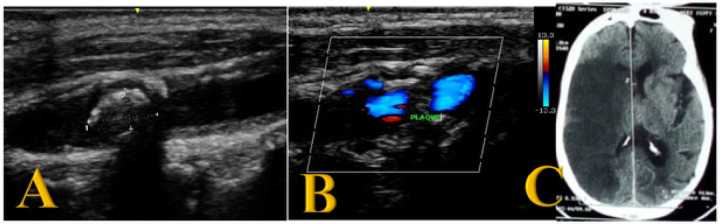
Severe carotid stenosis in a female with ischemic stroke Severe carotid artery stenosis in a 79-year-old female with a history of ischemic stroke. Sagittal ultrasound image (figure 2A) showing an atherosclerotic plaque with posterior shadowing in the Right common carotid artery. Figure 2B shows some color flow through the plaque. Axial CT image (Figure 2C) shows a large right sided ischemic infarct.

**Figure 3:**
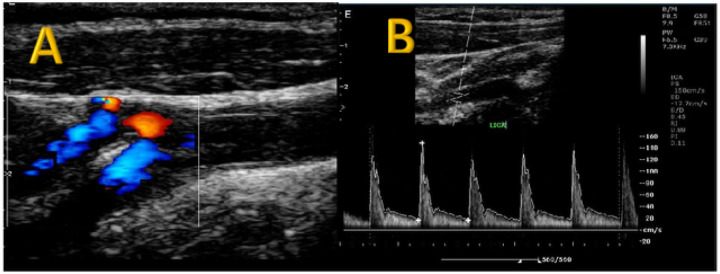
Moderate carotid stenosis Moderate carotid artery stenosis. Carotid ultrasound images of a 66-year-old female with long standing hypertension and diabetes and left sided ischemic infarct. Color doppler ultrasound (Figure 3A) sound shows irregular stenosed lumen of the left internal carotid artery. On spectral doppler, (Figure 3B), the peak systolic velocity was150cm/second, indicating moderate carotid artery stenosis.

The prevalence of significant carotid stenosis (more than 50% carotid stenosis) in study participants diagnosed with ischemic stroke was 12.5% (8/64). Moderate carotid stenosis was present in 4.7% (3/64) of the study participants. Fifty-six (87.5%) of the study participants had no significant stenosis. Samples of ultrasound and CT images for the participants with severe carotid stenosis are provided in Fig. 1 and Fig. 2. Moderate carotid artery stenosis ultrasound images are provided in Fig. 3A and Fig. 3B.

### Factors associated with thickened carotid intima media thickness

At multivariate analysis, the factors that were independently associated with thickened CIMT were age greater 60 years and high LDL. Participants above the age of sixty years were five times more likely to have a high CIMT than those below sixty years (aOR = 5.3, 95%CI: 1.2–14.0, *p* = 0.001). Participants with a elevated LDL were five times more likely to have a high CIMT (aOR = 4.2, 95%CI: 1.3–13.7, *p* = 0.14). (Table 4)

### Factors associated with carotid stenosis in ischemic stroke patients

At bivariate analysis, hypertension and elevated LDL cholesterol were associated with carotid stenosis. The associations were not sustained on multivariate analysis (Table 5)

## Discussion

### Carotid intima thickness

The prevalence of thickened CIMT (clinical atherosclerosis) was 31.5% in patients admitted with stroke. This is similar to a recent systematic review, meta-analysis and modelling data documenting the average global burden of high carotid intima media thickness to be 27% [19]. Other studies have documented a higher prevalence of carotid intima media thickness. A study done in Mulago hospital, a decade ago, documented the prevalence of atherosclerosis to be 46% among the stroke participants, a higher value than in our study and global estimates [26]. However, the CIMT cutoff values of this older study have not been documented in the published literature. Adeleye et al, in a study involving patients with known cardiovascular risk factors noted the prevalence of increased carotid intima media thickness to be 53% (cutoff for normal CIMT was 0.9 mm) [21]. Other studies that have documented higher CIMT denoting a high cardiovascular risk burden [36, 37]. The likely explanation for the higher prevalence of abnormal CIMT is due to the lower cutoff value used to define abnormal CIMT, thus, more individuals being classified as abnormal. We used a CIMT cutoff of 1mm or more to define thickened CIMT which has been reported to provide diagnostic sensitivity and specificity of CAD of 66% and 79% respectively [38]. We recommend future researchers to use a constant cutoff for comparability of study findings or present the various percentages of CIMT abnormalities based on the various cutoffs. A Malawian study revealed the prevalence of thickened carotid intima thickness to be 18% which is slightly lower than in the present study [39]. The difference could be due to our study having a higher mean age of participants (62 years), a higher prevalence of hypertension (88%) compared to the Malawian study. The prevalence in this study is higher than that documented by Ssinabulya *et al*, [40], whose study documented a prevalence of 18% in HIV positive patients. The patients in our study were older compared to the HIV study whose mean age was 37 years.

In our study, the prevalence of increased CIMT was higher in participants above 60 years and age was positively associated with increased CIMT. This agrees with most global studies that show increased CIMT, carotid plaque, and carotid stenosis being more common in older people than in younger people [19–21]. Elevated LDL cholesterol was also associated with an increased CIMT. Elevated LDL cholesterol is a known predictor for ischemic stroke and atherosclerotic cardiovascular events [21, 41, 42]. The infiltration and retention of apo*B* containing lipoproteins (the main structural protein in LDL) in the artery wall is a critical initiating event that sparks an inflammatory response and promotes the development of atherosclerosis in the arteries [41]. Thus, leading to an increase in CIMT.

### Carotid stenosis

The prevalence of carotid artery stenosis in study participants with a diagnosis of ischemic stroke was 12.5%. No patient with hemorrhagic stroke had significant stenosis (> 50%). Among the participants with ischemic stroke, 7.8% had severe carotid stenosis, 4.7% moderate stenosis and 87.5% had no significant stenosis. The prevalence of carotid artery stenosis in acute ischemic stroke patients is historically reported at 15–20% [43] with the prevalence being higher in the developed world and least in SSA. Findings in this study are lower than findings from Dutch study which found 18% of the participants with a diagnosis of ischemic stroke having more than 50% carotid stenosis [44]. The difference could be that patients in the Dutch study were older with a mean age of 70 years and most of them were smokers.

A large study involving ischemic stroke patients in Burkina Faso, similar to our study showed carotid stenosis was present in 23% of the study participants with severe carotid stenosis being present in 7% of the study participants [20]. Uganda and Burkina Faso are SSA, low-income countries experiencing epidemiological transitions with populations having a higher life expectancy and persistence of poorly controlled hypertension. A study at a tertiary health care hospital in Nigeria showed similar findings to our study. The study involved patients who were referred for cardiovascular screening and 16% had more than 50% carotid stenosis [30]. Similar findings are likely due to nearly similar risk profiles of the study population. A study at Kenyatta national hospital involving 126 patients admitted with a diagnosis of ischemic stroke found that the prevalence of carotid stenosis more than 50% was 5.6% with 1.6% of the patients having severe carotid stenosis and 4% having mild stenosis [45]. These findings are half those in the present study which could be due to the study participants having a lower mean age (59 years) in the Kenyan study. A hospital-based study in South Egypt showed significant carotid stenosis was present in 23% of the patients admitted with ischemic stroke [46]. The prevalence in this study was higher than in our study probably due to the higher prevalence of atherogenic risk factors with hypertension, hyperlipidemia, smoking, and diabetes mellitus being present in 97.7%,88.6%, 75% and 65% of the study participants.

Studies on the prevalence of carotid stenosis in stroke patients in Africa have yielded varying ranges of prevalence with older studies documenting absence or a very low prevalence of carotid stenosis [5, 29] while the more recent studies indicate a prevalence between six to thirty percent [20]. The prevalence in this study is higher than documented by Nakibuuka *et al,* [26], who found no evidence of carotid stenosis as a cause of stroke a decade ago. A study in Tanzania a decade ago involving 56 patients with stroke in which carotid doppler was performed yielded no significant carotid stenosis [27]. The high yield in this study could be that like in many LMIC, there is a growing burden of non-communicable diseases like hypertension in Uganda as part of an epidemiologic shift catalyzed by demographic and nutritional transitions[47]. The 2014 health demographic survey put the prevalence of hypertension, the commonest risk factor for stroke at 24% with most participants having poor blood pressure control [48]. It was inevitable that some of these individuals would be candidates for cerebrovascular events in the future with hypertension usually accelerating the atherosclerotic process. Uncontrolled hypertension was present in more than three quarters of the present study’s population.

Carotid artery disease is as common in African-American stroke patients as it is in Hispanics and whites, and as the burden and profile of stroke risk factors change in SSA, so it is likely that carotid artery disease will increase in the absence of adequate risk factor prevention [3].

In this study, hypertension had no significant association with carotid stenosis. A few other studies found no association between carotid stenosis and hypertension [49, 50]. Hypertension was also found to be the major risk factor of extracranial carotid atherosclerosis in most studies [19, 45, 51] due to its atherogenic effects. Diabetes, smoking, alcohol intake had no significant association with carotid artery stenosis in ischemic stroke patients in the present study. This is similar to study in Sri Lanka among ischemic stroke patients where no association was found between vascular risk factors (diabetes, smoking, alcohol intake) and carotid stenosis [52]. *Tan et al*, reported similar findings in their study of Taiwanese patients[50].

### Carotid plaques

The prevalence of carotid atherosclerotic plaques in the study was 26% with the majority being echogenic (60%). High risk echolucent plaques contributed to 40% of the plaque burden in participants with the atherosclerotic plaques. Most of the carotid atherosclerotic plaques were located at the carotid bulb. The global prevalence of carotid atherosclerotic plaques ranges from 13.2–21% [19]. A study carried out in Wakiso district of central Uganda among adults above the age of 60 years put the prevalence of carotid atherosclerotic plaques at 21% [53]. Studies in sub-Saharan Africa involving high risk cerebrovascular patients document a carotid plaque prevalence of between 15–30[20, 21]. In a meta-analysis involving 64 studies, high risk echolucent plaques were present in high risk patients with carotid stenosis [39].

Carotid studies in patients with symptomatic carotid artery stenosis have suggested an association between high-risk features of carotid plaques, in particular and an increased risk of recurrent ischemic stroke or transient ischemic attack[54]. Geroulakos *et al*, 2005, found echolucent plaques to be predominant in patients with symptomatic carotid disease. The preponderance of echolucent plaques in symptomatic patients with stenosis < 70 per cent supports the hypothesis that this type of plaque is unstable and tends to embolize [33]. In contrast, in patients without symptoms there is preponderance of echogenic plaques [33].

### Limitations

The evidence from this study is limited by its cross-sectional design and hospital-based setting. Other pro- atherosclerotic factors like chronic infections, diet, chronic inflammatory mediators, have not been evaluated in this study and may need to be evaluated in large multicenter studies.

## Conclusion

Compared with a decade ago there is a substantial increase in the burden of carotid atherosclerotic disease in patients admitted with stroke in Uganda i.e., **a**pproximately one in 10 Ugandans with stroke have significant carotid stenosis, a third have high CIMT, and quarter have atherosclerotic plaques. Age above 60 years, elevated LDL are associated with the high burden. Large multicenter further studies are needed to further profile carotid atherosclerotic disease risk factors in high-risk populations.

## Figures and Tables

**Table 1:** clinical and social demographic characteristics.

Variable	Frequency	Percentage
Age, mean (SD), Years	61.2 ± 13 years	

< 60 years	47	49.5
>=60	48	50.5

35–45	12	12.6
46–60	43	45.3
> 60	40	42.1

Gender (Male)	57	60

Smoking	19	20

Alcohol intake	56	58.9

Hypertension	73	76.8

Diabetes	19	20

High Triglycerides	26	27.4

Elevated LDL cholesterol	15	15.8

Low HDL cholesterol	89	93.7

Elevated total Cholesterol	24	25.3

Type of stroke		

Hemorrhagic	31	32.6
Ischemic	64	67.4

*LDL = Low density lipoproteins, *HDL = High density lipoproteins

**Table 2:** Carotid intima media thickness variables

Variable	Statistic/ Frequency(n)	Percentage
Average CMIT (IQR)	0.88 (0.75–1.07)	

*LCMIT (IQR)	0.87 (0.73–1.09)	

*RCMIT (IQR)	0.87(0.72–1.03)	

Thickened CIMT		

No	65	68.4
Yes	30	31.6

RCIMT thickness		
Thickened	31	32.6
Normal	64	67.3

LCIMT		

Thickened	34	35.8
Normal	61	64.2

*CIMT = carotid intima media thickness, *RCIMT = right common carotid intima media thickness, LCIMT = left common carotid intima media thickness, ICA = internal carotid artery, *IQR = interquartile range

**Table 3:** Frequency and location of atherosclerotic plaques

Variable	frequency(n/95)	Percentage (%)
Presence of Atherosclerotic plaques	25	26.1

Type of plaque	15	60
Echogenic	10	40
Echolucent		

Plaque location	2	8
CCA	16	64
Carotid bulb	7	28
ICA		

**Table 4:** Factors associated with carotid intima media thickness

Variable	All (N = 95) Freq (%)	Bivariate analysis		Multivariate logistic regression analysis		

		High CIMT (n= 30) n (%)	*p*-value	Adjusted odds ratio	95%Confidence Interval	*p*-value
Age, mean (SD), Years	61.2 (13)	67.3 (2.2)	0.002			

age > = 60	48 (50.5)	23 (39.7)	0.001	5.3	1.5–14.0	0.001

Male Sex	57 (60)	17 (29.8)	0.660			

Smoking	19 (20)	5 (26.3)	0.784			

Alcohol intake	56 (58.9)	17 (30.3)	0.829			

Hypertension	73 (76.8)	24 (32.8)	0.692			

High Triglycerides	26 (27.4)	6 (23.1)	0.329			

Elevated LDL	15 (15.8)	9 (60)	0.015	4.2	1.3–13.7	0.014

Low HDL	89 (93.7)	28 (31.4)	> 0.999			

High Cholesterol	24 (25.3)	9 (37.5)	0.612			

Diabetes	19 (20)	7 (36.8)	0.591			

**Table 5:** ; factors associated with carotid stenosis in ischemic stroke patients

Variable	Bivariate analysis			Multivariate logistic regression analysis		

	Ischemic stroke (n = 64)	Carotid stenosis (n = 8)	*p*-value	Adjusted odds ratio	95% confidence interval	*p*-value
Age, mean (SD), Years	63(13.1)	65(14.5)	0.649			

age > = 60	35(54.7)	5(14.3)	0.719			

Male Sex	39(60.9)	5(12.8)	> 0.999			

Smoking	13(20.3)	3(23.1)	0.343			

Alcohol intake	39(60.9)	6(15.4)	0.466			

Hypertension	43(67.2)	3(7.0)	0.010	1.5	0.47–5.08	0.474

High Triglycerides	21(32.8)	4(18.3)	0.422			

Elevated LDL	10(15.6)	3(30)	0.102	4.2	0.82–21.5	0.086

Low HDL	59(92.2)	7(11.9)	0.422			

High Cholesterol	17(26.6)	5(29.4)	0.429			

Diabetes	15(23.4)	2(13.3)	> 0.999			

## Abbreviations

CAD: Carotid atherosclerotic disease
CIMT: Carotid intima media thickness
NASCET: North American Symptomatic Carotid Endarterectomy Trial
ECST: European Carotid Surgery Trialists Group
